# Effect of supplementation of tomato juice on the oxidative stress of selected athletes

**DOI:** 10.1186/1550-2783-8-S1-P21

**Published:** 2011-11-07

**Authors:** L Ramaswamy, K Indirani

**Affiliations:** 1 Department of Nutrition & Dietetics. PSG College of Arts and Science, Coimbatore, India; 2 Department of Post Harvest Technology. Tamilnadu Agricultural University, Coimbatore, India

## Background

Oxidative stress occurs when there is an imbalance between production and scavenging of free radicals, thus compromising the cellular function and antioxidant status of the body. Athletes, who train competitively, experience more oxidative stress than other average individuals which not only causes damage to cells and DNA, but may also limit aerobic capacity. The present study was conducted to test the effect of tomato juice (lycopene) on the oxidative stress of athletes.

## Methods

Fifty male athletes involved in track events aged 20 - 25 years were selected for the study and divided into control (Group I) and experimental (Group II) of 25 each. A supplement containing 75ml of tomato juice (containing 10μg of lycopene), was consumed by the athletes of Group II after their morning training session for a period of 60 days. Venous blood samples were drawn immediately after training before supplementation and selected biochemical parameters were estimated. Again the samples were drawn after 60 days of supplementation to assess the changes in blood/serum indices and the results were compared with Group I. Twelve minute run test and step test were also conducted. The results were analysed using ANOVA and t test between control and experimental groups (p≤0.05).

## Results

The mean value of glutathione concentration (GSH) of Group II had increased significantly from 101.21 ± 46.41 mg/dl to 196.89 ± 35.11 mg/dl (t = 1.943, p ≤ 0.05) while that of Group I had decreased from 86.16 to 81.94 mg/dl (p > 0.05). The mean levels of glutathione peroxidase of Group II had increased significantly from 23.75 ± 9.01 μ/gHb to 41.03 ± 5.58 μ/gHb (t = 5.857, p ≤ 0.05) while the same had decreased significantly in Group I from 18.37 to 15.11 μ/gHb. The levels of TBARS (which is a measure of lipid peroxidation) had decreased significantly in Group II from 0.367 ± 0.111 to 0.197 ± 0.227 nmol/ml (t = 2.015, p ≤ 0.05) and in Group I from 0.446 ± 0.14 to 0.38 ± 0.152 nmol/ml (t = 1.139, p > 0.05). The distance covered in 12 minutes by the athletes of Group II increased significantly from 2305 ± 365.2m to 2734 ± 602.7m (t = 2.163, p ≤ 0.05), whereas the same had decreased in Group I from 2150.4 ± 471.5m to 2106.4 ± 365.2 m (p > 0.05) after the study period. The number of steps taken by Group II increased significantly from 31.91 ± 4.69 to 33.92 ± 4.57 (t = 5.057, p ≤ 0.05) while it had decreased in Group I. This indicates the efficiency of antioxidant defense system provided by the intake of tomato juice containing lycopene.

## Conclusion

It is concluded that lycopene in tomato juice has a beneficial effect on the oxidative stress on athletes and will improve their performance level when used as a supplement.

